# A Review of High-Power Semiconductor Optical Amplifiers in the 1550 nm Band

**DOI:** 10.3390/s23177326

**Published:** 2023-08-22

**Authors:** Hui Tang, Changjin Yang, Li Qin, Lei Liang, Yuxin Lei, Peng Jia, Yongyi Chen, Yubing Wang, Yue Song, Cheng Qiu, Chuantao Zheng, Xin Li, Dabing Li, Lijun Wang

**Affiliations:** 1 State Key Laboratory of Luminescence and Applications, Changchun Institute of Optics, Fine Mechanics and Physics, Chinese Academy of Sciences, Changchun 130033, China; tanghui21@mails.ucas.ac.cn (H.T.); yangchangjin20@mails.ucas.ac.cn (C.Y.); qinl@ciomp.ac.cn (L.Q.); leiyuxin@ciomp.ac.cn (Y.L.); jiapeng@ciomp.ac.cn (P.J.); wangyubing@ciomp.ac.cn (Y.W.); songyue@ciomp.ac.cn (Y.S.); qiucheng@ciomp.ac.cn (C.Q.); lidb@ciomp.ac.cn (D.L.); wanglj@ciomp.ac.cn (L.W.); 2 Daheng College, University of Chinese Academy of Sciences, Beijing 100049, China; 3 Peng Cheng Laboratory, No. 2, Xingke 1st Street, Shenzhen 518000, China; chenyy@ciomp.ac.cn (Y.C.); lix13@pcl.ac.cn (X.L.); 4 Jlight Semiconductor Technology Co., Ltd. No. 1588, Changde Road, Economic and Technological Development Zone, Changchun 130102, China; 5 State Key Laboratory on Integrated Optoelectronics, College of Electronic Science and Engineering, Jilin University, Changchun 130012, China; zhengchuantao@jlu.edu.cn

**Keywords:** semiconductor optical amplifier, high power, low noise, polarization insensitive, master oscillator power amplifier, LiDAR

## Abstract

The 1550 nm band semiconductor optical amplifier (SOA) has great potential for applications such as optical communication. Its wide-gain bandwidth is helpful in expanding the bandwidth resources of optical communication, thereby increasing total capacity transmitted over the fiber. Its relatively low cost and ease of integration also make it a high-performance amplifier of choice for LiDAR applications. In recent years, with the rapid development of quantum-well (QW) material systems, SOAs have gradually overcome the shortcomings of polarization sensitivity and high noise. The research on quantum-dot (QD) materials has further improved the noise characteristics and transmission loss of SOAs. The design of special waveguide structures—such as plate-coupled optical waveguide amplifiers and tapered amplifiers—has also increased the saturation output power of SOAs. The maximum gain of the SOA has been reported to be more than 21 dB. The maximum saturation output power has been reported to be more than 34.7 dBm. The maximum 3 dB gain bandwidth has been reported to be more than 120 nm, the lowest noise figure has been reported to be less than 4 dB, and the lowest polarization-dependent gain has been reported to be 0.1 dB. This study focuses on the improvement and enhancement of the main performance parameters of high-power SOAs in the 1550 nm band and introduces the performance parameters, the research progress of high-power SOAs in the 1550 nm band, and the development and application status of SOAs. Finally, the development trends and prospects of high-power SOAs in the 1550 nm band are summarized.

## 1. Introduction

The 1550 nm band high-power SOAs have important applications in optical communication, LiDAR, and other fields. Firstly, in the field of optical fiber communication, the 1550 nm band is the most important band for optical fiber transmission due to the combination of low loss and efficient amplifiers. With the rapid development of optical communication technology and the concept of an all-optical network, Tbit/s-level information transmission has been realized on a single fiber. Compared with erbium-doped fiber amplifiers (EDFAs), SOAs are small and low in cost. This makes them more suitable for optical communication in the future [1,2,3,4,5]. Although the larger linewidth of SOA results in fewer channels to accommodate in S-band and L-band compared to EDFA, the wider gain bandwidth of SOAs can provide new possibilities for expanding the bandwidth resources towards the S-band. By expanding new bandwidth resources, the channel transmission capacity can be further improved [5,6,7,8]. Secondly, in the field of space laser communication, the traditional solution is to use a semiconductor laser with a fiber amplifier above the Watt level as the space laser transmitter. However, its large size, high-power consumption, and poor radiation resistance can only meet the needs of short-term verification experiments. Semiconductor optical amplifiers are one to two orders of magnitude more resistant to radiation than fiber optic amplifiers, without any special improvements in radiation resistance [9,10,11,12,13,14]. High-power, low-cost, and integrated SOA devices are eminently suitable for carrying space loads for space laser communication. The United States, the European Union, and China are all actively conducting research in this area. Thirdly, in the field of LiDAR, the 1550 nm band laser is currently the most promising LiDAR band due to its low noise floor in the solar spectrum and a higher power threshold for human eye safety. To achieve the popularization of LiDAR, high-power optical amplifiers are essential [15]. However, current mainstream fiber amplifiers are expensive and bulky, hindering the further popularization of integrated LiDAR applications. Consequently, researchers have turned their attention to high-power semiconductor lasers and SOAs, in the hope of further reducing the price and volume of LiDAR devices and promoting the expansion of the LiDAR market using the unique advantages of semiconductor devices in terms of volume, weight, cost, and ease of integration [16,17].

In summary, the application prospects of high-power SOAs in the 1550 nm band are broad, with the main goal of researchers at present being the quest for high gain, high-saturation power, low noise, and low cost 1550 nm band SOAs.

In this study, the research progress of high-power SOAs in the 1550 nm band in recent years are discussed, including improvements and the promotion of their main performance parameters. The structure is arranged as follows: Section 2 presents key parameters commonly used for characterizing the performance of high-power SOAs and their definition, whereas improvements achieved with regard to the different performance parameters are detailed in Section 3. Developments of high-power SOAs in the 1550 nm band in recent years are indicated in Section 4, in view of different important applications of SOAs. Finally, development trends and prospects of SOAs are summarized in Section 5.

## 2. Theory: Performance Parameters of High-Power Semiconductor Optical Amplifiers

The parameters that measure the performance of high-power SOAs include the gain, saturation output power, noise figure (NF), and polarization-dependent gain, among others. Of these, gain is one of the most important parameters, especially for high-power SOAs. Although direct bandgap semiconductors have much higher gain than other laser media, they still cannot meet the high-power requirements in many cases. Consequently, researchers are still trying to achieve higher gain SOAs in various ways—including material systems [18,19], low-dimensional quantum structures [5,20], and waveguide structures [21,22]. In an ideal case without carrier loss, the gain coefficient of an SOA material can be expressed as follows [23,24,25]:

(1) $$\begin{array}{c} g\left(ћ\nu \right)=\frac{m_{r}^{*}e^{2}}{n_{r}c\varepsilon_{0}m_{0}^{2}\nu {ћ}^{2}L_{z}}\int_{0}^{\infty }dE_{t}{\left|\hat{e}*p_{cv}\right|}^{2}\frac{\gamma /\pi }{{\left[E_{h}^{e}+E_{t}-ћ\nu \right]}^{2}+\gamma^{2}}\times [f_{c}\left(E_{t}\right)-f_{v}\left(E_{t}\right)], \end{array}$$

(2) $$\begin{array}{c} \frac{1}{m_{r}^{*}}=\frac{1}{m_{e}^{*}}+\frac{1}{m_{h}^{*}}, \end{array}$$

(3) $$\begin{array}{c} f_{c}\left(E_{t}\right)=\frac{1}{1+\exp\left[\frac{E_{g}+E_{e}+\frac{m_{r}^{*}}{m_{e}^{*}}E_{t}-F_{c}}{k_{B}T}\right]}, \end{array}$$

(4) $$\begin{array}{c} f_{v}\left(E_{t}\right)=\frac{1}{1+\exp\left[\frac{E_{h}-\frac{m_{r}^{*}}{m_{h}^{*}}E_{t}-F_{v}}{k_{B}T}\right]}, \end{array}$$

where ћ denotes the reduced Planck’s constant, ν denotes the input optical signal frequency, e denotes the electronic power, $n_{r}$ denotes the refractive index, c denotes the speed of light in vacuum, $\varepsilon_{0}$ denotes the vacuum capacitance rate, $m_{0}$ denotes the electronic quality, $L_{z}$ denotes the quantum well thickness, ${\left|\hat{e}*p_{cv}\right|}^{2}$ denotes the momentum matrix element, γ denotes the refractive index spreading factor, $E_{t}$ denotes the composite center energy level, $E_{h}^{e}$ denotes the band-side leap energy, $E_{g}$ denotes the band gap energy, $m_{e}^{*}$ and $m_{h}^{*}$ denote the approximate masses of conduction band electrons and valence band holes, $E_{e}$ and $E_{h}$ denote the energy levels of electrons and holes, $F_{c}$ and $F_{v}$ denote the quasi-Fermi energy levels of electrons and holes, $k_{B}$ denotes the Boltzmann’s constant, and T denotes the reference temperature. It is evident from the factor $\left(f_{c}-f_{v}\right)$ that the gain exists only when the population inversion condition is satisfied.

For quantum well semiconductors, ${\left|\hat{e}*p_{cv}\right|}^{2}$ is polarization dependent. For example, for heavy cavities, ${\left|\hat{e}*p_{cv}\right|}^{2}$ can be expressed as follows [23]:

(5) $$\begin{array}{c} TE:{\left|\hat{e}*p_{cv}\right|}^{2}=\frac{3}{4}\left(1+\frac{E_{e}+\left|E_{h}\right|}{E_{e}+\left|E_{h}\right|+E_{t}}\right)*\frac{m_{0}}{6}E_{P}, \end{array}$$

(6) $$\begin{array}{c} TM:{\left|\hat{e}*p_{cv}\right|}^{2}=\frac{3}{2}\left(1-\frac{E_{e}+\left|E_{h}\right|}{E_{e}+\left|E_{h}\right|+E_{t}}\right)*\frac{m_{0}}{6}E_{P}, \end{array}$$

where $E_{P}$ denotes the energy parameters of matrix elements. It can be seen from Equations (5) and (6) that the gain of the quantum well for different polarization states can be changed by changing the conduction band electron and valence band hole energy.

Further, the gain of the SOA can be expressed as follows [26]:

(7) $$\begin{array}{c} G=\frac{e^{\left(\Gamma g-\alpha_{i}\right)L}\left(1-R_{1}\right)\left(1-R_{2}\right)}{{\left(1-e^{\left(\Gamma g-\alpha_{i}\right)L}\sqrt{R_{1}R_{2}}\right)}^{2}+4e^{\left(\Gamma g-\alpha_{i}\right)L}\sqrt{R_{1}R_{2}}{\sin\left[\frac{\pi \left(\nu -\nu_{0}\right)}{{∆\nu }_{L}}\right]}^{2}}, \end{array}$$

where *L* is the length of the SOA active region, Γ denotes the mode field confinement factor, $\alpha_{i}$ denotes the loss coefficient, $R_{1}$, $R_{2}$ denote the reflectance of both ends, $\nu_{0}$ denotes the center frequency of the gain spectrum, and ${∆\nu }_{L}$ denotes the longitudinal mode interval.

It is evident from Equations (1) and (7) that a direct way to increase the gain is to increase the density of states involved in the radiation transition, and energy band engineering is one of the most effective ways. By constructing low-dimensional quantum structures, such as QWs, the density of states of the material can be distributed in steps with energy. The density of states in the QW sub-bands are independent of energy. Consequently, the electrons in the sub-bands can participate in the transition with the same probability, thereby greatly increasing the density of states participating in the radiation transition. Another method is to appropriately extend the length of the active region to increase the gain and to reduce the reflectivity of the end facet to reduce the loss. This can be achieved by improving the waveguide structure and end-facet coating.

The saturation output power is another important parameter of high-power SOAs. With increasing high electron injection density, the gain saturation in the semiconductor gain medium is reached, its gain deviating from the linear growth region, and the growth slowing down or even decreasing. The saturation output power can be defined as the output power corresponding to a 3 dB decrease in gain, expressed as follows [25]:

(8) $$\begin{array}{c} P_{3dB}=\frac{h\nu A\eta_{0}ln2}{\tau \Gamma dg/dN}, \end{array}$$

where A denotes the cross-sectional area of the SOA active layer, $\eta_{0}$ denotes the output coupling coefficient, τ denotes the carrier lifetime, and dg/dN denotes the differential gain.

The principal reason for the gain saturation is that the high-density electron injection enhances the shielding effect of the optical field and reduces the stimulated radiation recombination in the gain medium. This leads to a gradual imbalance in the consumption and replenishment of many carriers during the stimulated emission process, inhibiting gain increases. If high-saturation output power is desired, the saturation of the SOA output power using the spontaneous radiation power in the gain medium should be minimized. A common method of increasing the saturation output power is to increase the cross-sectional area of the SOA active layer to reduce the optical power per unit area. Reducing the mode field limiting factor and the differential gain can also increase the saturation output power.

The SOA NF represents the degradation of the signal-to-noise ratio caused by the signal passing through the SOA, which directly affects the signal quality after the SOA amplification. Its main source is the random fluctuation of light intensity and phase generated by the interaction of electrons and photons in the gain medium. In most application fields, especially in the field of optical communication, the aim is to reduce the SOA NF as much as possible. For example, reducing the NF of all amplifiers in a link increases the optical signal-to-noise ratio at its end by 1 dB. This, again, allows users to reduce the launch power into the link by 1 dB. This can effectively alleviate the nonlinear effects in the fiber, and its economic benefits are clear [1,27,28]. A low-noise SOA is also important in the master-oscillator power amplifier (MOPA), enabling low-noise, high-power, narrow-linewidth coherent sources.

Generally, the SOA NF can be set as the ratio of the signal-to-noise ratio of the shot-noise limited input signal and the signal-to-noise ratio of the amplified output signal, which can be expressed as follows [25]:

(9) $$\begin{array}{c} NF=10\mathrm{lg}\left(F\right)=10\mathrm{lg}\left(\frac{{SNR}_{input}}{{SNR}_{output}}\right), \end{array}$$

where ${SNR}_{input}$ and ${SNR}_{output}$ denote the signal-to-noise ratio at the input and output of the SOA, respectively.

The main way to reduce the SOA noise figure is to reduce the signal-spontaneous emission beat noise. A common method is to improve the low-dimensional quantum structure to reduce the signal-spontaneous emission beat noise factor by using a strained QW or QD structure.

Polarization-dependent gain refers to the SOA gain difference in different input signal polarization states. Compared with the polarization insensitivity advantage of EDFAs due to their fiber structure, SOAs often exhibit a gain difference of 5–8 dB between the TE and TM modes without special designs, which can also affect the effective gain bandwidth. Consequently, in applications such as optical-fiber communication, the SOA should be as polarization insensitive as possible. The polarization-dependent gain (∆G) of the SOA can be defined as the ratio of the maximum gain of the TE mode to the minimum gain of the TM mode, expressed as follows [25]:

(10) $$\begin{array}{c} ∆G=\frac{G_{max}^{TE}}{G_{min}^{TM}}=\left[\frac{{\left(1+\sqrt{R_{1}R_{2}}G_{s}^{TM}\right)}^{2}}{{\left(1-\sqrt{R_{1}R_{2}}G_{s}^{TE}\right)}^{2}}\right]\exp\left[\left(\Gamma_{TE}-\Gamma_{TM}\right)gL\right], \end{array}$$

where $R_{1}$, $R_{2}$ denote the reflectance of both end facets, $G_{s}^{TE},\;G_{s}^{TM}$ denote the one-way gains of the TE and TM modes, respectively, $\Gamma_{TE},\;\Gamma_{TM}$ denote the light field confinement factors of the TE and TM modes, respectively, g denotes the material gain, and L denotes the SOA active area length.

Based on Equation (10), there are three main factors affecting SOA polarization-dependent gain—that is, (1) in the gain medium, the radiation of the TE mode caused by the degenerate process is much larger than that of the TM mode, the gain difference further expanding as the current increases; (2) the waveguide structure of the SOA is often rectangular, $\Gamma_{TE}$ often being larger than $\Gamma_{TM}$ in such a waveguide structure; and (3) there is a difference between the loss of the TE mode and the loss of the TM mode at the end of the SOA (that is, the difference between the $R_{1}$ and $R_{2}$ values), which also affects the SOA gain of the two modes to a certain extent. Consequently, to realize the SOA polarization insensitivity, researchers often adjust the SOA polarization-dependent gain by increasing the thickness of the active layer or changing the low-dimensional quantum structure [29,30,31].

In addition to the above performance parameters, SOAs also have other performance parameters, including the ASE optical 3 dB bandwidth, linewidth broadening, gain disturbance, gain-recovery speed, linewidth enhancement factor, and four-wave mixing frequency, amongst others. These parameters have different requirements based on different functional necessities.

With the continuous development of optical communication and optical detection technology, the market demand for optical amplifiers is increasing, with low-cost and high-performance optical amplifiers having excellent market prospects. To adapt to the use of optical-fiber communication transmission and LiDAR detection, SOAs are increasingly developing high power, wide-gain spectrum, low noise, polarization insensitivity, and high integration features.

## 3. Research Progress of High-Power Semiconductor Optical Amplifiers in the 1550 nm Band

### 3.1. High Power

**Figure 1:**
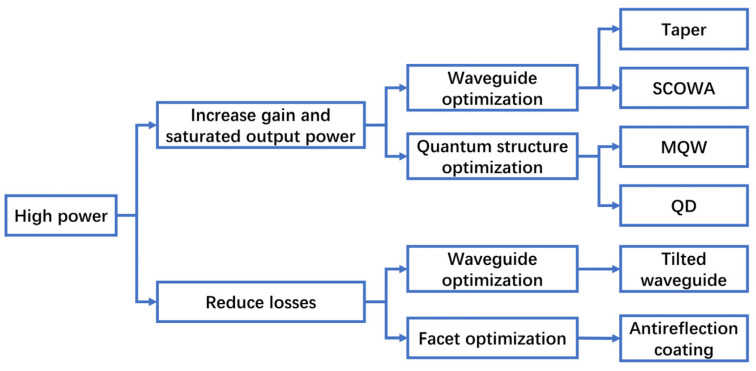
Main technical roadmap of high-power SOAs.

As shown in Figure 1, a high-power SOA focuses on increasing the gain and saturation output power and reducing the loss. Although increasing the injection current is the most intuitive way to increase the output power, eventually the SOA output power will tend to be saturated and will no longer increase. Too high of a current will generate too much heat, making the SOA unstable and increasing the load on the cooling system. It also makes the SOA adversely affected by Auger non-radiative recombination. Therefore, the SOA is generally made to work under the condition of saturation output power to prolong the lifetime of the device.

**Figure 2:**
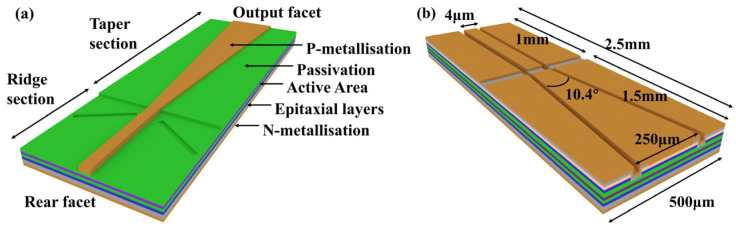
(**a**) Schematic structure of a tapered SOA. (**b**) Two-stage amplified optical cavity structure.

In terms of increasing the gain and saturation output power, the main methods are to improve the shape of the waveguide and the low-dimensional quantum structure. It is common to achieve a gain of more than 13 dB and a saturation output power of more than 20 dBm. To improve the waveguide structure, one can extend the length of the waveguide appropriately, or the width of the waveguide, to increase the area of the gain region, thereby increasing the gain. However, if the waveguide is extended too much, it can saturate the second half of the waveguide, resulting in losses. Additionally, if the waveguide is too wide, it can easily cause multi-mode lasing, which makes the SOA unstable. Moreover, an excessively wide waveguide will also bring about additional losses. Consequently, in follow-up research, tapered structures have been used to improve SOA output power and stability [32,33,34,35]. In an ideal tapered waveguide, the light mode propagates along the length of the cone, continuously expanding due to diffraction. When the tapered region is pumped with current, this mode can be amplified, resulting in high optical output power. Keeping the taper angle constant, the longer the waveguide, the greater the mode spread and the higher the achievable output power [36]. A wider taper angle allows the SOA to achieve higher power and improved beam quality, but it also means using a higher drive current [37,38]. In 2020, Ogrodowski, L’s research group reported a non-uniform QW tapered SOA, which achieved a gain bandwidth of over 100 nm by optimizing the QW thickness [39]. As shown in Figure 2a, the device design uses a 6° taper angle and a 5 mm long taper cavity, enabling it to operate in the 780–1010 nm spectrum at a power of 4–5 W. In 2021, Wang LJ’s research group reported a two-stage amplifying optical cavity structure, as shown in Figure 2b, obtaining a gain of 13.8 dB, a saturation output power of 600 mW, and a 3 dB gain bandwidth of 70 nm [26]. The device design uses a 1 mm long and 4 µm wide ridge waveguide and a 1.5 mm long tapered waveguide with a tilt angle of 10.4°. Fabricated using standard i-line lithography with micron-level precision, high power, wide-gain bandwidth, and linewidth broadening as low as 1.15× were achieved. In 2022, Leisher’s research group reported a diffraction-limited 1550 nm tapered SOA [40]. The device realized a new record of over 3.0 W saturation output power, with a diffraction-limited beam quality (M2~1.2) nearly at the maximum current of 12 A. The length of the device was 4.5 mm, including a 1.5 mm MO ridge section and a 3 mm PA ridge section. This showed us a new way to achieve saturation output power through SOA that was previously only possible with EDFA. The high-power characteristics of the tapered SOA afford great potential for low-cost, high-power SOA fabrication. However, the light spot at the output of the tapered structure can be overly scattered, making it difficult to couple the emitted radiation into the fiber—that is, excessive coupling loss can limit the practical use of conical SOAs.

**Figure 3:**
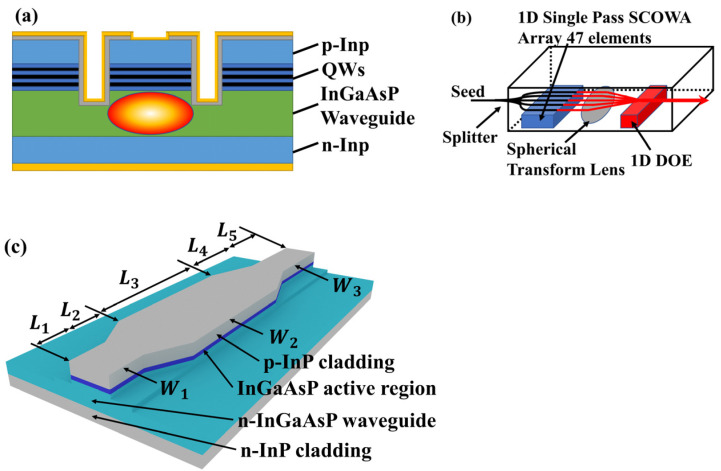
(**a**) Schematic cross-section of a slab-coupled–optical-wave amplifier (SCOWA) waveguide. (**b**) Schematic structure of SCOWA Array. (**c**) Schematic structure of VC-SCOWA.

The slab-coupled–optical-waveguide amplifier (SCOWA) is another waveguide SOA solution. Different from the traditional ridge waveguide SOA structure etched to the upper waveguide layer, the SCOWA places the QW-layer above the waveguide layer and uses deep etching to etch through the entire QW layer, as shown in Figure 3a. This design enables the SCOWA to have larger light spot cross-sectional dimensions and lower single-mode output loss than conventional ridged waveguides, thus enabling higher saturation output power [41,42,43,44]. Moreover, the SCOWA can achieve near-circular spatial light output, which can be more efficiently coupled into single-mode fibers without the assistance of lenses [45,46,47,48].

In 2005, Juodawlkis PW’s research group reported a SCOWA of InGaAsP-InP QWs, which achieved a fiber-to-fiber gain of 13 dB and saturation output power of +31 dBm in the 1.5 μm band [49]. The device length was 1 cm, which expanded the gain area, and the longer chip length was conducive to heat dissipation. In 2011, Juodawlkis PW’s research group reported a new type of variable confinement SCOWA (VC-SCOWA), as shown in Figure 3c [50]. A ridge waveguide design of varying widths is used to further reduce the QW optical confinement factor, resulting in higher output saturation power. The device divides the ridge waveguide into two layers—that is, the lower ridge is fixed at 5.8 μm, and the upper ridge is narrower at the output end, which is 4 μm. Moreover, it gradually widens in the middle section to the same width as the lower ridge width to improve the input and output fiber couplings as much as possible. The experiment showed that the output saturation power of the VC-SCOWA was nearly 2 dB higher, reaching +27.6 dBm, compared to the SCOWA with a fixed ridge width of 5.8 μm. It also exhibited an unsaturated gain of 21.1 dB and a low noise figure of 5.3 dB. In 2012, Smith GM’s research group reported an amplifier array using 47 SCOWA bundles, as shown in Figure 3b, which achieved a total output power of 57 W using a single SCOWA seed power of 50 mW [22]. The length of a single SCOWA in the array is 5 mm, and it can achieve a continuous wave output of 1.5 W using 50 mW of seed power. Finally, a phase controller is used for beam combining. This solution opened new ideas for traditional ridge waveguide SOAs to further improve their output power. The deeply etched waveguide structure of the SCOWA exhibits many advantages in terms of gain and saturation output power, but it can also complicate the SCOWA preparation process.

**Figure 4:**
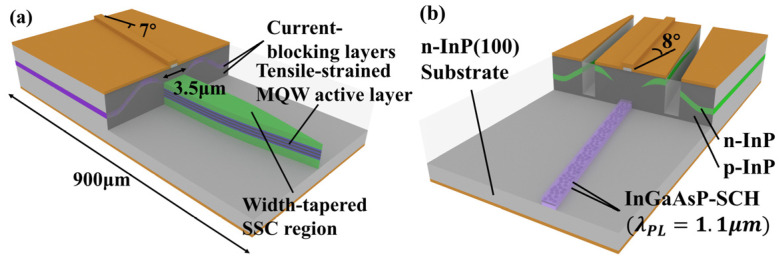
**Figure 4**. (**a**) Schematic structure of a tensile-strained multi-quantum-well (QW) SOA chip. (**b**) Schematic structure of a tilted SOA.

For improved low-dimensional quantum structures, the gain coefficient and saturation output power can be effectively improved by stacking multiple layers of QWs or QD structures [18,51]. In a multi-quantum-well (MQW) SOA, thin QWs can be used to achieve a flat and wide-gain spectrum. However, to stabilize the gain spectrum at high power, it is necessary to control the thickness of the QW so as not to be too thin to maintain a large energy separation [52,53,54]. In 2005, Morito K’s research group reported a broadband multi-QW SOA, which achieved a gain of 15 dB over a 3 dB gain bandwidth of 120 nm, a saturation output power of 19.6 dBm, and a low NF of 4.5 dB [5]. The device used three 5 nm InGaAsP thin QWs sandwiched between four InGaAsP barriers, and the width of the active waveguide was 3.5 μm. In 2006, Morito K’s research group reported an SOA based on a thin-strained multi-QW structure. As shown in Figure 4a, a light spot size converter is integrated at the output end, achieving +20 dBm fiber-coupled saturation output power and 6 dB NF [54]. The thin and wide-edged layer effectively increases the saturation output power of the SOA. The active layer of the strained MQW structure comprises a tensile-strained barrier and an unstrained QW. It can compensate for the gain difference between the TE and TM modes of the material to a certain extent, reducing the polarization-dependent gain of the device. QD materials have many advantages—such as high gain and a high-gain recovery speed—but the saturation output power is lower than that of QW materials. Consequently, the output power is often improved by stacking multiple layers or synchronously improving the waveguide structure—such as by combining it with tapered waveguides. A tapered SOA using a multi-layered QD structure can further increase the output power while maintaining the advantages of the QD structure [20]. In 2005, Akiyama T’s research group reported a QD ultra-wideband SOA using a slanted waveguide structure, as shown in Figure 4b. Using the ultrafast gain response of QDs and the ability to improve the Fermi level at large current densities, the device realized a 25 dB gain, a 22 dBm saturation output power, and a 5 dB NF [55]. To enable the gain bandwidth to cover the 1.5μm band, the research group used InAs Stranski–Krastanow QDs on an InP(100) substrate. In 2018, Technion’s research group reported a high gain and low temperature sensitivity QD SOA [56]. The device realized a static gain level at the peak gain spectrum of about 22 dB up to at 50 °C. It also realized distortionless amplification of a single 28 Gbit/s signal and amplification of two channels separated by 2 nm. Its stable temperature characteristics brought more advantages to the device in the application of complex integrated silicon photonics circuits. The QDs had less lattice mismatch, and, thus, they could be made larger. It is evident that improvement of the low-dimensional quantum structure effectively improves the performance of the SOA, but the processing difficulty and costs increase exponentially.

### 3.2. Low-Noise Figure (NF)

A major disadvantage of the SOA compared to the EDFA is that the NF is nearly 3 dB higher, which greatly limits the application of SOAs in wavelength division multiplexing (WDM) systems. Even though high-power SOAs have cost advantages, it is still difficult to further popularize SOAs in the field of optical communication due to them accommodating too few channels in the C-band and L-band (which are mainly used nowadays). A common low-noise SOA can suppress NF to around 5 dB. There are three primary ways to reduce NF—that is, an improved low-dimensional quantum structure, improved waveguide structure, and cascading [57,58,59].

Firstly, using a special QW structure or using a QD structure can effectively suppress the noise of an SOA [60]. In 2005, Morito K’s research group reported a multi-QW SOA with an NF of 4.5 dB, and its minimum NF was close to 3.6 dB [5]. In the experiment, thin multiple QWs with low loss were used to achieve a high-saturation output power and low NF, while also achieving a 15 dB gain and a 19.6 dBm saturation output power over a 3 dB gain bandwidth of 120 nm. In 2021, the Yu, SQ research group reported a 1550 nm low NF SOA design with a detuned material at the input [61]. The device kept a low NF, below 6.5 dB, over the C and L bands. The experiment showed that by inserting a detuned material on one-fourth to one-half of the SOA length, the NF can be reduced by 1.5 dB. In 2010, Hasegawa H’s research group theoretically and experimentally verified the influence of the number of QWs and waveguide structures on the NF of a multi-QW SOA, and they designed a compressively strained multi-QW SOA with 5 QWs and an NF of 3.7 dB [62]. The QD SOA is a better low-noise SOA due to its lower threshold and higher ion number inversion relative to QWs and bulk materials [63,64,65,66,67].

Secondly, improving the waveguide structure—for example, by adopting the inclined waveguide structure—can reduce the loss, end-facet reflection, and noise [27,68]. As mentioned above, Morito K’s research group realized a low-noise hybrid QW SOA with an NF of 6 dB using a straight waveguide structure with a tilt of 7° [54]. In 2011, Juodawlkis PW’s research group reported a SCOWA with a minimum NF of 4.5 dB [69]. It considered both high output power and low noise, achieving a small signal gain of 12.8 dB and a saturation output power of 0.8 W. It is evident that the SCOWA has enormous potential in the field of high-power, low-noise SOAs.

Thirdly, the multi-stage cascaded SOA is another way of effectively reducing noise. For example, a multi-stage power supply can be formed using electrode groove isolation on the waveguide, or a multi-section SOA can be cascaded to improve performance. Moreover, various studies have shown that an uneven power supply on a straight waveguide could further improve the saturation output power of the straight waveguide SOA and reduce the noise. Consequently, we can divide the waveguide into three sections—that is, the front, middle, and rear—using electrode isolation grooves.

**Figure 5:**
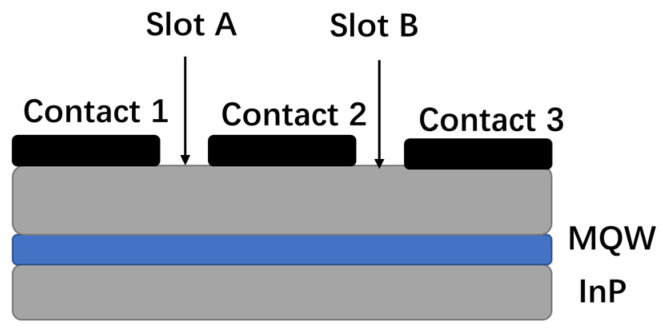
Schematic structure of bulk SOA.

The middle section can be used as the main gain amplification area, with the bias voltage of the front and rear sections being adjusted to reduce noise and increase the saturation output power [8,70,71,72]. In 2013, Carney, K’s research group reported a multi-contact SOA with an NF of 3.8 dB, as shown in Figure 5 [8]. The device can be divided into three independent power supplies to realize control of the carrier distribution in the SOA. The experiments show that the NF of the SOA can be further reduced by adjusting its internal carrier density. In 2017, Duill SPO’s research group built a simplified numerical model of a multi-section SOA suitable for the simulation of an optical communication system. This simulation model determined that a multi-section SOA could provide a 3 dB greater input power dynamic range compared with that of a single-section SOA, proving that the multi-segment SOA had better noise and linearity performance [73].

In recent years, although SOAs have been able to reduce the NF to near EFDA levels in the ways described above, there is still a huge gain gap between the two. EDFAs can provide more than 50 dB of gain at an NF of 4 dB [74], and can even monitor devices for gain control. This is much higher than the highest power SOAs now. However, we can still expect SOAs to improve significantly over time. SOAs can more easily achieve more than 60 nm of 3 dB gain bandwidth, which is a more difficult challenge for EDFAs [7,75]. Therefore, SOAs still have great potential in areas such as optical communications.

### 3.3. Polarization Insensitivity

**Figure 6:**
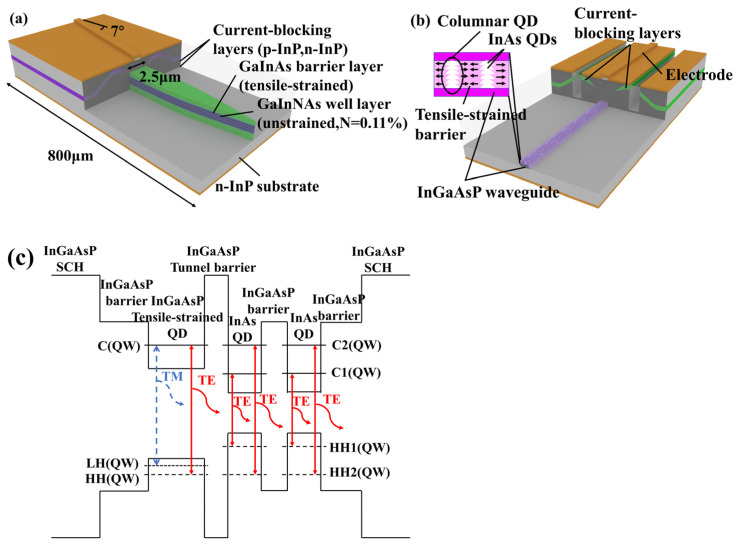
(**a**) Schematic structure of the GaInNAs–GaInAs MQW-SOA. (**b**) Schematic structure of the CQD-SOA. (**c**) Schematic structure of QDs coupled to QWs.

Today’s 1550 nm polarization-insensitive SOA can generally achieve a polarization-dependent gain within 1 dB, and some can be lower than 0.5 dB. There are two main ways of achieving SOA polarization insensitivity—that is, increasing the thickness of the active layer and changing the low-dimensional quantum structure. From Equation (10), it is evident that increasing the thickness of the active layer can increase the confinement factor of the TM mode, thereby reducing the polarization-dependent gain [30,76]. However, the thickening of the active layer also affects the reliability and saturation output power of the SOA, so it is not practical to increase the active layer thickness blindly. From Equations (1), (5) and (6), we can see that the gain of the quantum well on TE and TM modes can be changed by the tensor-strain quantum well structure. Therefore, the gain difference between the TE and TM modes due to the waveguide structure can be compensated for using the strained QW structure, which can make the gain of the device in TE and TM modes similar and realize polarization insensitivity [6,77]. In 2008, Morito K’s group reported a polarization-insensitive SOA based on GaInNAs–GaInAs strained multiple QWs. As shown in Figure 6a, the device redshifts the gain band by 30 nm by adding nitrogen to the strained barrier MQW layer, achieving a gain tilt of less than 1.2 dB and a polarization dependence of less than 0.8 dB over a total gain bandwidth of 90 nm in the 1510–1600 nm band gain [19]. At 300 mA, the device had a gain of 12.6 dB, a saturation output power of 12.8 dBm, and an NF of 6.5 dB. In 2009, Huang Lirong’s research group adopted the QD-coupled tensile-strained QW design methodology, as shown in Figure 6c, to realize a polarization-insensitive SOA [31]. Tensile-strained QWs not only provide gain for the TM mode in the edged layer but also can be used as a carrier injection layer for QDs. Compared with the general QD SOA, the design of QW-coupled QDs effectively improves the gain of the device, reducing the threshold current and improving the temperature stability. In 2020, Zali’s research group performed an in-depth analysis and designed a strained-barrier InGaAs/InGaAs MQW SOA [78]. Mulation displayed that the device had a low polarization-dependent gain below 3 dB. The research group’s in-depth numerical analysis of the strained quantum well structure has considerable reference value. In recent years, it has been found that the gain of the active region in the TM mode can be effectively enhanced by adjusting the QD structure. Its polarization properties can be controlled by adjusting the aspect ratio of the QD structure and the strain of the lateral barrier, thereby enabling polarization insensitivity of the device [79,80,81,82]. In 2012, Yasuoka, N’s research group reported a polarization-insensitive SOA in the 1.5 μm band using strain-controlled columnar QDs [83]. As shown in Figure 6b, the QDs have a high aspect ratio and a strain-controlled side barrier, achieving a gain factor of 8 dB, a polarization-dependent gain of 0.4 dB, and a saturation output power of 18.5 dBm. In 2017, Farmani, A’s group reported a polarization-dependent gain of less than 0.1 dB in the 1550 nm band by appropriately thickening the SOA active layer of the QDs [84]. The polarization insensitivity, saturation output power, and gain are all realized by the excellent saturation output power performance of the QDs.

It can be seen from Table 1, Table 2 and Table 3 that, with the development and maturity of technologies such as SCOWAs, QDs, and strained QWs, the performance of high-power SOAs has been considerably improved. Nowadays, high-power SOAs in the 1550 nm band can meet the requirements of high-gain and high-saturation output power, and feasible technical solutions in terms of low noise and polarization insensitivity have been explored. It can be foreseen that, shortly, high-power SOAs could achieve high output power, low noise, and polarization insensitivity, becoming the first choice for high-performance and low-cost optical amplifiers in the fields of optical communication and detection.

## 4. Discussion: Development and Application of High-Power Semiconductor Optical Amplifiers in the 1550 nm Band

### 4.1. Master-Oscillator Power Amplifier (MOPA)

**Figure 7:**
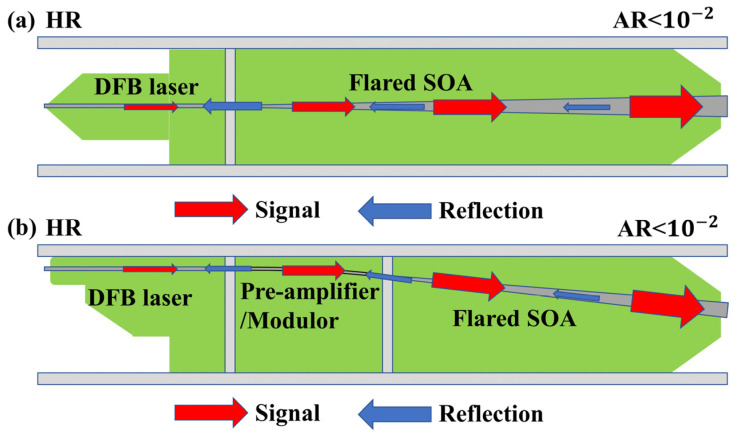
Schematic structure of the master-oscillator power amplifier (MOPA). (**a**) Two-segment structure. (**b**) Three-segment structure.

One of the most common high-power SOA applications in the 1550 nm band is to combine it with a 1550 nm distributed feedback laser (DFB). This laser—which achieves its high output power through the high gain of the SOA—is called the MOPA [86,87,88,89]. To further improve the output power, the MOPA often uses a tapered SOA. As shown in Figure 7, the MOPA is a two-segment structure including a DFB laser and a high-power amplifier, or a three-segment structure including a DFB laser, a modulation part, and a high-power amplifier [90,91,92,93,94]. Faugeron M’s research group showed that the instability of the MOPA device comes primarily from two factors—that is, DFB laser interference by the light reflected onto the DFB laser from the device coupling and the mode competition between the entire cavity defined by the two end facets and the DFB laser. To improve the MOPA stability, a key factor is to reduce the reflectivity of the device section. Consequently, a certain tilt angle is often introduced in the modulation part and the tapered SOA part to reduce the device reflectivity [95,96,97,98]. In 2018, Pham C’s research group reported a three-stage MOPA with a tilted structure, which achieved an output power of 380 mW [99,100]. The device consisted of a shallow-ridged DFB laser, a shallow-ridged modulator, and a tapered SOA with the same vertical structure. The device modulator adopted a curved waveguide structure, and the tapered SOA adopted a 7° tilt-angle structure. The device could achieve stable operation within 500 mA, and the side-mode suppression ratio (SMSR) was more than 40 dB. The MOPA has great prospects in LiDAR and space optical communication applications due to its small size, light weight, and good radiation resistance. To compete with fiber and solid-state lasers, MOPAs need to have stable outputs of more than 0.5 W. Consequently, the SOA needs to improve its gain and saturation output power as much as possible, while exhibiting relatively low-noise performance and a small line width.

### 4.2. Laser Communication

**Figure 8:**
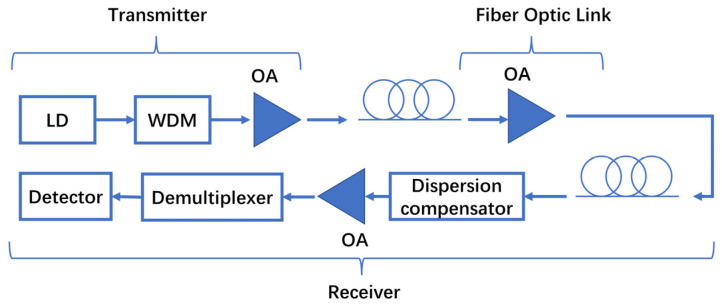
Schematic structure of fiber-laser communication.

Laser communication comprises communication technologies using different transmission media, such as optical fiber communication, atmospheric laser communication, and space laser communication. Fiber laser communication is now the hottest. Its commonly used communication bands are the 1064 nm, 1310 nm, and 1550 nm bands. The gain bandwidth coverage of the high-power SOA in the 1550 nm band is one of them. As shown in Figure 8, in WDM systems, we need a post-amplifier to compensate for the insertion loss of the external modulator required for high-rate modulation, an in-line amplifier to compensate for fiber loss in the fiber optic link, and a pre-amplifier to improve the receiver sensitivity [25]. In the remaining two communication technologies, especially in space laser communication, optical amplifiers need to consider the influence of environmental factors such as the high radiation in space. In 2018, Zhao, HW’s research group reported an InP-based integrated optical transmitter integrated with the SOA, which achieved error-free signal transmission over an equivalent distance of 180 m at a data rate of up to 3 Gbps. Under conditions of forward error correction, the distance could be extended to 300 m [28]. In 2020, Renaudier, J’s research group reported an optical-fiber transmission system with a bandwidth of more than 100 nm, which was realized using an SOA. Through this WDM system, the research group realized the transmission of 250 channels with an interval of 50 GHz and a total capacity of 115.9 Tb/s, opening up new possibilities for future intercity long-distance optical-fiber communication [101]. Compared to the EDFA fiber-laser communication system, which has been able to achieve several kilometers of communication distance, the SOA fiber-laser communication system still has many shortcomings, especially the transmission distance, which is short. But the SOA offers much higher radiation resistance, a smaller size, a lighter weight, low power consumption, wide-gain bandwidth, and many other advantages, which can hopefully lead to highly integrated and low-cost laser communication modules. To achieve farther information transmission, it is necessary to overcome the low power problems of traditional SOAs and rapidly promote the development of Watt-level high-power SOAs [1,102,103,104,105,106,107,108,109]. To meet the requirements of WDM in optical communication, SOAs should have a wide and flat gain spectrum, low polarization-dependent gain, and good working stability. Moreover, in different locations, SOAs have different focus requirements. As a post-amplifier at the transmitting end, or as an in-line amplifier in a fiber optic link, the SOA focus on having high saturation output power and low NF in order to meet the power requirements of long-distance communication, and they do not bring in too much noise. As a pre-amplifier at the receiving end, the SOA focuses on having low NF to minimize the effects of noise when restoring the signal. Every performance improvement of the SOA can improve the optical communication distance along with its contingent economic benefits. With the increasingly fierce inter-country competition in space, optical communication is becoming increasingly important. All countries attach great importance to this and to the development of high-power SOAs, hoping to develop SOAs that meet their communication needs.

### 4.3. LiDAR

**Figure 9:**
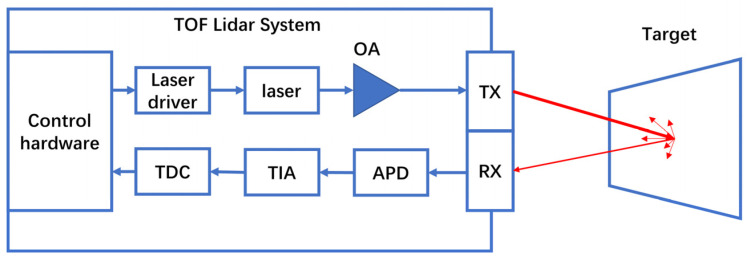
Schematic structure of pulsed LiDAR.

Compared with microwave radar, LiDAR exhibits high resolution, strong anti-interference abilities, and small size, and it is lightweight, making it a better choice for high-performance perception systems [15,110,111]. Consequently, LiDAR is gradually replacing millimeter-wave radar and becoming the core of a new generation of perception systems. The bottleneck of LiDAR market expansion is the distance measurement and radar cost. For example, automotive LiDAR needs to have a low enough cost under the premise of ensuring a 200 m range in order to gain a foothold in the extremely price-sensitive automotive detector market [112,113,114,115,116]. The commonly used time-of-flight (TOF) LiDAR principle is shown in Figure 9. Here, the optical amplifier is used primarily to amplify the pulsed optical signal at the transmitting end. Consequently, the amplifier must be able to adapt to the working state of the high-frequency pulse and have the largest possible saturation output power. Currently, mainstream solutions use the EDFA as the amplifier of the transmitting end—which is relatively expensive—and high-power EDFAs can be bulky, wasting the valuable structural space of the car. Compared with the combination of traditional solid-state laser and EDFAs, the MOPA is more compact, has lower power consumption and cost, and is expected to become the laser-light source of choice in new generations of radar [16,17,89]. In 2022, the Liang M’s research group reported a MOPA for LiDAR systems, using a two-stage design of a DFB laser and a tapered SOA. The saturation output power was close to 1 W, and the line width reached 200~300 kHz using a stable power supply. The research group then performed lens-to-fiber coupling and butterfly packaging on the MOPA, achieving a net coupling efficiency of 70% [117]. Based on its low cost, the MOPA has been successfully commercialized in applications such as wind-farm radars that do not require high accuracy but are in great demand [118]. However, for the highly competitive automotive LiDAR field, the MOPA still has vast room for improvement. For TOF technology, the MOPA’s key parameters are its peak power and short pulse. For frequency modulated continuous wave (FMCW) technology, the MOPA focuses more on achieving continuous tunability. The SOA is extremely competitive, whether it is used as a MOPA amplifier or directly used as an optical amplifier for LiDAR instead of the EDFA. Generally, it is suitable for reducing the cost and volume as much as possible while increasing the laser power to achieve longer distance ranging, although there are still many performance indicators that need to be optimized [119,120,121]. In TOF technology, the SOA needs to exhibit higher gain and saturation output power, as well as a faster gain-recovery speed, to meet the amplification requirements of high-frequency–pulsed-laser signals. In FMCW technology, the SOA must have a wider gain bandwidth and reduced linewidth broadening to ensure that the optical signal can change smoothly within a certain wavelength range and exhibit good coherence. In today’s optical phased array (OPA) LiDAR, the overall loss of the transmitting end can be relatively large. To achieve a longer detection range and a larger detection angle, a frequency-modulation light source with higher power and a narrower linewidth is required.

With the development of optical communication and detection technologies, the scale of silicon photonic integrated circuits (PICs) is rapidly increasing. As one of the key components of a PIC, the SOA has attracted much attention. Although silicon, as an indirect bandgap semiconductor, has low luminous efficiency, it has a very mature complementary metal-oxide semiconductor process in the field of microelectronics. Moreover, thanks to the extremely low price of silicon, the cost of chips is being further reduced. Silicon-based integrated SOAs have a variety of options, including bonding III–V SOAs to silicon wafers [122,123]; hybrid integration of III–V SOAs and PICs, such as flip-chip–reflective-semiconductor optical amplifiers on a silicon chip to form an external cavity laser [124,125]; epitaxy of heavily doped germanium material on silicon [126]; III–V heteroepitaxy on silicon; and more [127]. In 2016, Davenport, ML’s research group reported a heterojunction silicon/III–V family of high power and high-gain SOAs. It achieved a non-saturated gain of 25 dB at the highest gain design, a gain bandwidth of 65 nm, and a saturation output power of 16.25 dBm [128]. The device used direct bonding of III–V SOAs to the silicon waveguide, leaving air spacers on both sides of the waveguide. Moreover, the transition of III–V/Si was conducted using a tapered structure, which achieved a low loss of 0.5 dB and a reflection of less than 45 dB. In 2019, Van Gasse K’s group reported a III–V-on-silicon SOA array using direct bonding [129]. It consists of five SOAs of the same cross-section but differing lengths, ranging from 0.95 to 1.85 mm, of which the 1.45 mm SOA shows the best performance. It achieved a small signal gain of 27 dB, a saturation output power of 17.24 dBm, and an NF of less than 8 dB. Nowadays, the SOA has been widely used in PICs—such as for power compensation of each channel in the OPA array—improving the detection distance of the OPA LiDAR; integrated with tuned lasers for WDM transmission to optimize the output linewidth and SMSR; and integrated into the optical path of the WDM receiver, reducing the number of external amplifiers at the receiving end. However, silicon-based integrated SOAs still face many problems. The thermal expansion coefficient mismatch between InP and Si is large, so the thermal stability of the device can be relatively poor, and silicon has a higher refractive index than InP, so the silicon waveguide layer of the device tends to be narrower, resulting in additional scattering losses. Consequently, silicon-based integrated SOAs need to further optimize the bonding surface process to meet the application requirements of PICs in the field of optical communication and optical detection. A silicon-based integrated SOA requires a smaller line width, lower NF, and higher operating stability.

### 4.4. Optical Gyroscope

**Figure 10:**
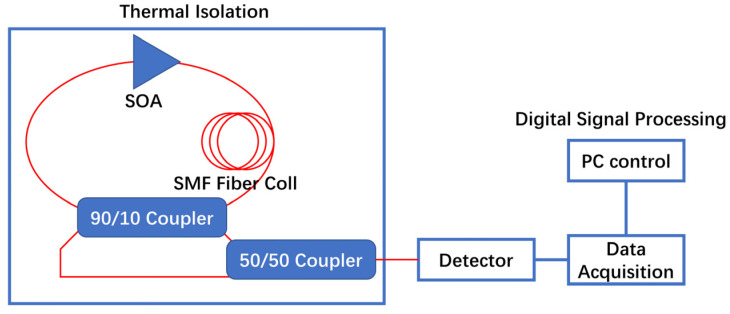
Schematic structure of the ring-laser gyroscope.

Optical gyroscopes are inertial sensors based on the Sagnac effect for measuring the angular velocity of an axis of rotation. The two main types of optical gyroscopes are ring-laser gyroscopes (RLGs) and fiber-optic gyroscopes (FOGs). To further improve the accuracy of high-precision FOGs, it is necessary to increase the output power of the ASE light source. However, this can cause the relative intensity noise to dominate and affect the measurement results. Consequently, the SOA is often used as the preamplifier of the optical gyroscope. When the SOA works in the saturation region, it can suppress the relative intensity noise while amplifying the light-source signal and adjusting the feedback to reduce noise and further improve the detection accuracy of the FOG [130,131,132,133]. In 2008, Inagaki, K’s research group successfully detected the rotation speed of the earth using an active RLG comprising an SOA and fiber-ring resonator. This was the largest active RLG at the time, proving the feasibility of SOAs for FOGs in achieving precise measurements [134]. In 2016, Shebl, A’s group reported an optical gyroscope of a hybrid semiconductor fiber-ring laser comprising a standard single-mode fiber and an SOA, as shown in Figure 10. It uses standard single-mode fiber as the ring resonator, an SOA as the optical gain medium, and a fiber coupler to achieve feedback [130]. Through the advantages of small size, low coupling between counter-propagating waves, and uniform broadening of the SOAs, it achieved a good linear scale factor of laser gyroscopes, a dynamic range of up to 40°/s, and a sensitivity of 0.2°/s. In fiber-optic ring gyroscopes, the SOA must amplify multiple longitudinal modes, and must exhibit a flat and wide-gain spectrum and lower noise to improve the gyroscopic sensitivity. SOAs working in the deep saturation region can effectively suppress the relative intensity noise of the optical gyroscope, and can reduce the relative intensity noise by about 5 dB/Hz [135,136], although the SOA still has some gaps in coupling efficiency and connection robustness. Excessive loss will also affect the sensitivity of the optical gyroscope. However, the suppression effect of the SOA on relative intensity noise and its smaller size also make it more applicable in optical gyroscopes.

## 5. Conclusions

In the continuous development of optical communication and detection technology, optical amplification technology is one of the important fulcrums, such that the market demand for high-power SOAs is increasing by the day. The 1550 nm band is the most used band for optical communications, and it is the most promising laser band for mainstream applications for future LiDAR popularization. Consequently, high-power SOAs in the 1550 nm band could be a hot spot for future industrialization, with market demand driving continuous technology development. To date, solutions such as the SCOWA and MOPA have shown enormous potential with deepening research. In the future, we should further improve the output power of high-power SOAs in the 1550 nm band, considering their performance indicators—such as stability and noise—to meet the needs of industrialization. With in-depth research and technological progress, as well as the maturity of QD theory and related technologies, the next generation of high-power SOAs in the 1550 nm band should be able to address the previous problems of high noise, polarization sensitivity, and poor stability. Furthermore, through their unparalleled cost and integration advantages, high-power SOAs will play a pivotal role in the future development of optoelectronics. We have reason to believe that the continuous development of the optoelectronics industry, the development of new technologies, and the continuous emergence of new demands will inevitably drive the sustainable development and continued success of SOA technology.

## Figures and Tables

**Table 1:** The high-power SOAs discussed in this paper.

References	Time	Research Group	Structure	Gain	3 dB Gain Bandwidth	Saturation Output Power
Ref. [85]	2003	Borghesani, A	Strained MQW	17 dB	-	16.5 dBm
Ref. [53]	2003	Morito K	Strained MQW	19 dB	-	17 dBm
Ref. [52]	2005	Morito K	Strained MQW	15 dB	-	22 dBm
Ref. [5]	2005	Morito K	Strained MQW	15 dB	120 nm	19.6 dBm
Ref. [55]	2005	Akiyama T	Tilted QD	25 dB	90 nm	22 dBm
Ref. [49]	2005	Juodawlkis PW	SCOWA	13 dB	>115 nm	31 dBm
Ref. [54]	2006	Morito K	Strained MQW	10.4 dB	-	20 dBm
Ref. [19]	2008	Morito K	MQW	12.6 dB	90 nm	12.8 dBm
Ref. [68]	2011	Juodawlkis PW	SCOWA	12.8 dB	>100 nm	29 dBm
Ref. [50]	2011	Juodawlkis PW	VC-SCOWA	21.1 dB	120 nm	27.6 dBm
Ref. [22]	2012	Smith GM	SCOWA	-	-	31.7 dBm
Ref. [83]	2012	Yasuoka N	QD	8 dB	>80 nm	18.5 dBm
Ref. [56]	2018	Eyal, O	QD	20 dB	-	9.6 dBm
Ref. [26]	2021	Wang LJ	Tapered MQW	13.8 dB	70 nm	27.8 dBm
Ref. [40]	2023	Leisher	Tapered MQW	-	-	>34.7 dBm

**Table 2:** The low-noise SOAs discussed in this paper.

References	Time	Research Group	Structure	Noise Figure
Ref. [85]	2003	Borghesani, A	Strained MQW	6 dB
Ref. [53]	2003	Morito K	Strained MQW	7 dB
Ref. [70]	2004	Saini SS	Cascade	5.6 dB
Ref. [52]	2005	Morito K	Strained MQW	5.7 dB
Ref. [5]	2005	Morito K	Strained MQW	4.5 dB
Ref. [55]	2005	Akiyama T	Tilted QD	5 dB
Ref. [54]	2006	Morito K	Strained MQW	6 dB
Ref. [19]	2008	Morito K	MQW	6.5 dB
Ref. [62]	2010	Hasegawa H	MQW	3.7 dB
Ref. [68]	2011	Juodawlkis PW	SCOWA	5.5 dB
Ref. [71]	2011	Bradley, AL	Cascade	5 dB
Ref. [8]	2013	Carney K	Cascade	3.8 dB
Ref. [60]	2015	Mazzucato	Bulk	<5 dB
Ref. [61]	2021	Yu, SQ	MQW	6.5 dB

**Table 3:** Polarization-insensitive SOAs discussed in this paper.

References	Time	Research Group	Structure	Polarization-Dependent Gain
Ref. [85]	2003	Borghesani, A	Strained MQW	0.8 dB
Ref. [53]	2003	Morito K	Strained MQW	0.2 dB
Ref. [52]	2005	Morito K	Strained MQW	0.5 dB
Ref. [54]	2006	Morito K	Strained MQW	0.6 dB
Ref. [19]	2008	Morito K	MQW	0.8 dB
Ref. [83]	2012	Yasuoka N	QD	0.4 dB
Ref. [84]	2017	Farmani A	QD	0.1 dB
Ref. [78]	2020	Zali	Strained MQW	<3 dB

## Data Availability

The data that have been used are confidential.
